# Effect of sarcopenia and paraspinal muscle morphology on conservative treatment outcomes in lumbar spinal stenosis: a prospective cohort study

**DOI:** 10.3389/fragi.2026.1913710

**Published:** 2026-08-26

**Authors:** Zhangmeng Xu, Tao Xu, Jing Guo, Jinshun Zou, Jiajia Luo, Fan Chen, Xin Zhang, Ji Wu

**Affiliations:** 1 Department of Cervicodynia/Omalgia/Lumbago/Sciatica, Sichuan Province Orthopedic Hospital, Chengdu, Sichuan, China; 2 Department of Rehabilitation, Sichuan Province Orthopedic Hospital, Chengdu, Sichuan, China

**Keywords:** cross-sectional areas, lumbar spinal stenosis, mediation effect, paraspinal muscles, sarcopenia

## Abstract

**Introduction:**

The impact of sarcopenia on postoperative outcomes in lumbar spinal stenosis (LSS) is well documented, but its role in conservative treatment remains unclear. In this prospective cohort study, we examined the association between sarcopenia and outcomes after conservative treatment in elderly LSS patients and explored whether paraspinal cross-sectional area (CSA) and fat percentage mediate this relationship.

**Methods:**

Elderly LSS patients undergoing conservative treatment at Sichuan Provincial Orthopedic Hospital (June 2023–May 2025) were enrolled. Sarcopenia was diagnosed according to the criteria of the Asian Working Group for Sarcopenia. The primary outcome was >50% improvement in the Japanese Orthopedic Association (JOA) score at discharge. Missing data were imputed with random forest. Candidate predictors were screened using univariate logistic regression (*p* < 0.1) and selected using the least absolute shrinkage and selection operator (LASSO) regression. Sensitivity analyses comprised Firth’s penalized correction for sparse data, subgroup analyses, and examination of individual sarcopenia components [appendicular skeletal muscle mass index (ASMI), grip strength, and gait speed] in relation to treatment outcome. Mediation analysis with bootstrap resampling evaluated total paraspinal CSA and mean fat percentage as mediators.

**Results:**

Among 463 patients, 89 (19.2%) had sarcopenia. Sarcopenic patients had significantly smaller paraspinal CSA and greater fat infiltration than non-sarcopenic patients. After adjustment for sex, age, and albumin, sarcopenia remained independently associated with poor discharge outcome (OR = 0.21, 95% CI: 0.12–0.38, *p* < 0.001). Subgroup analyses showed consistent effects across age, sex, body mass index (BMI), albumin, and stenosis severity strata (all *p* for interaction >0.05). Among individual components, ASMI and grip strength independently predicted poor outcomes, whereas gait speed did not. Mediation analysis demonstrated that total paraspinal CSA mediated 74.64% of the effect (*p* = 0.012), whereas mean fat percentage showed no significant mediation (*p* = 0.710). Sensitivity analyses confirmed the robustness of these findings against missing data, model specification, and sparse data bias.

**Conclusion:**

Sarcopenia is independently associated with worse short-term outcomes after conservative treatment in older LSS patients, with this association statistically mediated by reduced paraspinal CSA.

## 1 Introduction

Lumbar spinal stenosis (LSS) is a degenerative spinal disorder characterized by narrowing of the spinal canal, lateral recess, or nerve root canal, which compresses the dural sac or nerve roots. Its main clinical manifestations include neurogenic claudication and low back and leg pain (Katz et al., 2022). With the acceleration of population aging, the global number of patients with LSS has exceeded 100 million, making it a major disease burden affecting the quality of life and functional independence of elderly people (Jensen et al., 2020; Salo et al., 2026).

Although surgical decompression is currently the recognized effective treatment for severe LSS, a considerable proportion of patients, especially those with milder symptoms or contraindications to surgery, choose or require conservative treatment. Conservative strategies mainly encompass supervised exercise rehabilitation, acupuncture, physical therapy, oral analgesia, and interventional therapies such as epidural steroid injections (Ammendolia et al., 2022; Zhu et al., 2024; Katz et al., 2022). However, treatment responses are highly heterogeneous across cohorts partly because previous studies have used variable criteria for defining satisfactory outcomes, ranging from MCID in pain scales (Minetama et al., 2024) and improvements in walking distance (Deer et al., 2019) to self-reported symptom relief (Schneider et al., 2019). Clinical data suggest that approximately 30%–50% of patients with mild-to-moderate symptoms experience meaningful improvement (Jensen et al., 2021), while another study indicates that 15%–30% of patients may experience gradual deterioration and eventually require surgical intervention (Shipley, 2008). A large longitudinal study of more than 140,000 patients further reported that 25.3% underwent surgery within 5.7 years of initial diagnosis (Naidu et al., 2024). Long-term follow-up studies further illustrate the divergent trajectories of conservatively managed LSS: approximately one-third of patients experience sustained relief, one-third remain stable, and one-third show progressive functional decline over a decade (Minamide et al., 2013). This prognostic heterogeneity underscores the urgent need to identify reliable predictors of treatment response.

Multiple investigations have attempted to identify baseline predictors associated with conservative treatment failure, including clinical parameters such as prolonged symptom duration, severe baseline neurogenic claudication (Tsubosaka et al., 2018), concurrent degenerative spondylolisthesis, multi-radicular nerve impairment, and psychological comorbidities, as well as smoking and obesity (Bagley et al., 2019; Davison et al., 2021). Notably, most existing prognostic research on conservative management of LSS primarily focuses on demographic, clinical, and conventional radiological morphological indicators. Emerging evidence suggests that muscle-related characteristics may represent promising prognostic markers (Jin and Wi, 2025), yet these muscle-related factors remain insufficiently validated in non-surgical LSS cohorts. Collectively, these findings indicate that early identification of key modifiable and non-modifiable factors influencing conservative treatment outcomes is of great clinical significance for optimizing treatment strategies and implementing precise patient stratification.

Sarcopenia is an age-related syndrome characterized by progressive loss of skeletal muscle mass, strength, and function and is particularly common in patients with chronic diseases (Yuan and Larsson, 2023). In recent years, the relationship between sarcopenia and spinal degenerative diseases has received increasing attention. Studies have shown that patients with lumbar degenerative disease who have sarcopenia exhibit significantly poorer functional improvement, pain relief, and quality of life after surgery than those without sarcopenia (Chua et al., 2022; Regev et al., 2026). More recent studies further indicate that paraspinal muscle loss is also an important predictor of postoperative outcomes in LSS patients (Ekşi et al., 2025; Narayanan et al., 2025). Although the influence of sarcopenia and paraspinal muscle morphology on postoperative outcomes in LSS patients has been well documented, whether they play similarly important roles in patients undergoing conservative treatment remains to be fully elucidated.

Based on the above background, this prospective cohort study was designed to systematically evaluate the epidemiological characteristics of sarcopenia and its impact on in-hospital outcomes in elderly LSS patients receiving conservative treatment and to further explore the mediating role of paraspinal muscle morphological parameters in the relationship between sarcopenia and prognosis using mediation analysis, aiming to provide evidence-based support for risk stratification and individualized management of conservative treatment for LSS.

## 2 Materials and methods

### 2.1 Study design and participants

This was a single-center prospective cohort study. Consecutive elderly patients with LSS who received conservative treatment at Sichuan Provincial Orthopedic Hospital from 1 June 2023 to 31 May 2025 were enrolled. The study protocol was approved by the Ethics Committee of Sichuan Provincial Orthopedic Hospital (No.: KY2023-008-01). All patients provided written informed consent, and all procedures were conducted in accordance with the principles of the Declaration of Helsinki. The inclusion criteria were as follows: (1) age ≥60 years; and (2) primary diagnosis at admission was LSS. The exclusion criteria were as follows: (1) acute lumbar fracture; (2) isthmic spondylolisthesis; (3) previous lumbar surgery; (4) intraspinal tumor, infection, or tuberculosis; (5) severe cardiac, pulmonary, hepatic, or renal insufficiency or malignancy; and (6) severe cognitive impairment or psychiatric disorder that prevented cooperation with assessments.

### 2.2 Diagnostic criteria

#### 2.2.1 Diagnostic criteria for LSS

Diagnosis was based on the North American Spine Society (NASS) diagnostic criteria (Kreiner et al., 2013), combining clinical symptoms, physical signs, and imaging features, after excluding vascular claudication, tumors, and other disorders that could cause similar manifestations (Webb et al., 2024).

Regarding clinical symptoms, patients should have typical neurogenic intermittent claudication, which can be relieved by forward flexion, bending, or squatting and aggravated by extension; low back pain, radicular pain in specific dermatomes, and paresthesia (numbness and heaviness) may also be present. On physical examination, a positive lumbar extension test or the presence of corresponding muscle weakness, sensory loss, or abnormal tendon reflexes is supportive. Imaging criteria (on axial magnetic resonance imaging, MRI): for imaging diagnosis, all cases underwent lumbar MRI. Stenosis severity was graded using the Schizas LSS grading classification, which is based on the morphology of the dural sac on axial T2-weighted images (Schizas et al., 2010). All images were independently reviewed and graded by two senior spine surgeons who were blinded to clinical information. Disagreements were resolved through consensus discussion.

#### 2.2.2 Diagnostic criteria for sarcopenia

The stepwise approach of the 2019 Asian Working Group for Sarcopenia (AWGS) criteria was followed (Chen et al., 2020).

Gait speed assessment: All patients underwent a 6-m linear walking test. Those with a gait speed ≤1 m/s proceeded directly to muscle mass measurement; those with a gait speed >1 m/s required further grip strength measurement.

Grip strength assessment: Grip strength of the dominant hand was measured using an electronic hand dynamometer (Xiangshan Electronic Hand Dynamometer, China). Low grip strength was defined as <28 kg for men and <18 kg for women. Patients with low grip strength proceeded to muscle mass measurement.

Muscle mass assessment: Whole-body dual-energy X-ray absorptiometry (Hologic Discovery A, United States) was used to measure appendicular skeletal muscle mass, analyzed using Hologic APEX software (version 4.0). The appendicular skeletal muscle mass index (ASMI) was calculated as ASMI = ASM (kg)/height^2^ (m^2^). Low muscle mass was defined as ASMI <7.0 kg/m^2^ for men and <5.4 kg/m^2^ for women.

Diagnosis of sarcopenia: Both low muscle mass and either low grip strength or slow gait speed were required.

### 2.3 Treatment protocol

All patients received standardized conservative treatment, which was based on the “Guideline for Integrated Traditional Chinese and Western Medicine Diagnosis and Treatment of Lumbar Spinal Stenosis (2023)” and mainly included traditional Chinese medicine therapy, physical therapy, and oral medications.

For traditional Chinese medicine therapy, patients received daily acupuncture and tuina. Acupuncture points mainly included Shenshu (BL23) and Dachangshu (BL25) with a needle depth of 0.5–1 cun and retention for 20 min; tuina using rolling and kneading techniques to relax the lumbar muscles was performed once daily. For physical therapy, patients received daily medium-frequency electrotherapy and infrared irradiation. Medium-frequency electrotherapy (2,000 Hz) was applied to the lumbosacral region for 20 min per session; infrared irradiation was applied to the lumbosacral region for 15 min per session to promote local blood circulation and tissue repair.

For oral medications, all patients received routine mecobalamin (0.5 mg three times daily) for nerve nutrition. In cases of significant pain [visual analog scale (VAS) score ≥4], celecoxib (200 mg twice daily) was added as a short-term nonsteroidal anti-inflammatory drug (NSAID). NSAIDs were contraindicated in patients with a history of peptic ulcer, gastrointestinal bleeding, or hepatic or renal insufficiency. For such patients, the treating physicians could prescribe alternative agents at their discretion, including acetaminophen, or neuropathic pain agents, such as pregabalin or gabapentin.

### 2.4 Data collection

1. General data and laboratory indices: Age, sex, living status (living alone or not), body mass index (BMI), serum albumin, blood urea nitrogen, creatinine, C-reactive protein (CRP), white blood cell count, neutrophil count, platelet count, lymphocyte count, and hemoglobin.
2. Lifestyle and comorbidities: Smoking history (continuous or cumulative smoking for ≥6 months); alcohol consumption history (according to the Chinese Dietary Guidelines: men drinking >1 time/week with >25 g alcohol each time, women >15 g, for ≥6 months); hypertension (during hospitalization, three separate measurements with systolic pressure >140 mmHg or diastolic pressure >90 mmHg, or previous diagnosis); diabetes mellitus (fasting glucose ≥7.0 mmol/L or random glucose ≥11.1 mmol/L, or previous diagnosis); hyperlipidemia (total cholesterol ≥5.2 mmol/L, or triglycerides ≥1.7 mmol/L, or LDL-C ≥3.4 mmol/L, or a previous diagnosis).
3. Paraspinal muscle parameters: All patients underwent lumbar MRI using a 1.5-T superconducting scanner (Siemens, Germany) with sequences including sagittal T1WI, T2WI, and axial T2WI. Two radiologists blinded to the grouping independently outlined muscle borders on axial T2-weighted images at the stenotic segment disc level using ImageJ software, measuring the cross-sectional area (CSA) (cm^2^) of the bilateral psoas, erector spinae, and multifidus muscles. Fat percentage was quantified using the adjacent subcutaneous fat signal as a reference threshold; intramuscular fat CSA was automatically segmented, and the fat infiltration percentage for each muscle was calculated as follows: (intramuscular fat area/total muscle CSA) ×100%. To assess interobserver reliability, intraclass correlation coefficients (ICCs) were calculated based on a two-way random-effects model for absolute agreement (Koo and Li, 2016). The final values for each parameter were calculated as the average of the two independent measurements.
4. Outcome measures: VAS scores at admission and discharge, Japanese Orthopedic Association (JOA) scores at admission and discharge, and JOA scores at 1 month and 3 months after discharge were recorded.

The JOA score improvement rate was calculated as follows: (follow-up JOA − admission JOA)/(29 − admission JOA) ×100%. An improvement rate >50% at discharge was defined as effective and served as the primary outcome (Alicioglu et al., 2012). A JOA improvement rate >50% at 1 month after discharge was used as a secondary outcome for sensitivity analysis.

### 2.5 Statistical analysis

All analyses were performed using R (version 4.5.1, R Foundation for Statistical Computing, Vienna, Austria). Normality of continuous variables was assessed using skewness and excess kurtosis: variables with absolute skewness <1 and absolute excess kurtosis <2 were considered approximately normally distributed (Kim, 2013). Normally distributed continuous variables are presented as mean ± SD and compared using independent t-tests; non-normally distributed variables are presented as median (25th and 75th percentiles, i.e., Q1 and Q3), and between-group comparisons are performed using the Mann–Whitney U test. Categorical variables are presented as count (%) and compared using the chi-square test or Fisher’s exact test, as appropriate. Ordinal variables, such as Schizas grade, were analyzed using the Mann–Whitney U test. All tests were two-sided, and *p* < 0.05 was considered statistically significant.

Missing data: Random forest imputation was applied. Imputation adequacy was assessed by comparing variable distributions before and after imputation.

Variable selection: Univariate logistic regression was first performed for each variable (*p* < 0.1 as a candidate threshold). Candidates were then entered into least absolute shrinkage and selection operator (LASSO) regression with 10-fold cross-validation. Although LASSO itself performs variable selection, preliminary univariate screening was used as a conservative strategy to reduce the candidate pool, improve model stability, and maintain interpretability (Chatterjee et al., 2025). The optimal λ was selected using the 1-SE rule, and variables with non-zero coefficients were retained for the final multivariable logistic model. Effect sizes for sarcopenia were reported from progressively adjusted models.

Mediation analysis: Because sarcopenia strongly correlated with paraspinal muscle parameters, the sum of psoas, erector spinae, and multifidus CAS was calculated as the total paraspinal area (cm^2^), and the mean of their fat percentages was calculated as mean paraspinal fat (%). Each composite variable was evaluated as a mediator using bootstrap analysis (5,000 resamples) to estimate the average causal mediation effect (ACME), average direct effect (ADE), and proportion mediated. The selection of total paraspinal CSA and mean fat percentage as candidate mediators was based on biological plausibility, literature support, and statistical feasibility: CSA and fatty infiltration are the most extensively studied MRI-based paraspinal phenotypes in LSS research and represent the two fundamental dimensions of paraspinal muscle health directly linked to spinal stability and functional outcomes (Lawan et al., 2025).

Sensitivity analyses: (1) complete case analysis excluding patients with any missing data; (2) multivariable linear regression using length of hospital stay as a continuous outcome; (3) Firth’s penalized maximum likelihood estimation with both fully adjusted and simplified models (adjusting for age, sex, or albumin separately) for the 1-month and 3-month outcomes, where sparse data bias was a concern due to the small number of events in the sarcopenia group (only 8 and 14 good outcomes, respectively) (Heinze and Schemper, 2002); (4) subgroup analyses stratified by sex, age, BMI, albumin, Schizas grade, gait speed, and grip strength to examine the consistency of the sarcopenia effect across strata; and (5) exploratory analyses of the independent associations of each sarcopenia diagnostic component (ASMI, gait speed, and grip strength) with discharge outcome.

## 3 Results

### 3.1 Patient disposition and missing data imputation

A total of 496 LSS patients were initially enrolled; 33 were excluded due to missing data for sarcopenia diagnosis, admission JOA score, or discharge JOA score, resulting in a final analysis cohort of 463 patients.

Among the included patients, eight variables (sarcopenia, sex, age, length of hospital stay, admission JOA, discharge JOA, ASMI, and gait speed) had no missing values. For the remaining variables, the missing proportion was mostly <10%, except for JOA at 1 month (17.06%) and JOA at 3 months (29.81%) after discharge. There were no statistically significant differences in the distribution of any variable before and after imputation, indicating that the random forest imputation was adequate and preserved the original data distribution (Supplementary Table S1). Stratified comparisons by sarcopenia status also showed that the imputation maintained consistent distributions within both the non-sarcopenia and sarcopenia groups (Supplementary Tables S2a,b).

### 3.2 Comparison of clinical characteristics between sarcopenia and non-sarcopenia groups

Among the 463 patients, 89 (19.2%) had sarcopenia, and 374 (80.8%) did not. Normality assessment showed that all continuous variables were approximately normally distributed, except for CRP (Supplementary Table S3). As shown in Table 1, the sarcopenia group had a higher proportion of female patients, older age, higher prevalence of hypertension, lower BMI, lower levels of total protein and albumin, higher CRP levels, significantly lower ASMI, slower gait speed, and lower grip strength than the non-sarcopenia group. There were no significant differences between groups in living status, history of diabetes, hyperlipidemia, smoking, alcohol consumption, renal function indices, or main stenosis level. The distribution of Schizas grades also did not differ significantly between groups (*p* = 0.441). Regarding analgesic use, a higher proportion of patients in the sarcopenia group required analgesics (74.2% vs. 66.0%), although this difference was not statistically significant (*p* = 0.142). The overall distribution of analgesic types differed significantly between groups (*p* = 0.007): celecoxib was the most commonly prescribed agent in both groups (55.1% vs. 57.5%), while the use of gabapentin (6.7% vs. 1.1%) and pregabalin (7.9% vs. 5.3%) was higher in the sarcopenia group, and acetaminophen use was also slightly more frequent in sarcopenic patients (4.5% vs. 2.1%).

**TABLE 1:** Comparison of characteristics between patients with and without sarcopenia.

Variable	Total (n = 463)	Non-sarcopenia (n = 374)	Sarcopenia (n = 89)	*p*-value
Sex, n (%)	​	​	​	0.026
Male	194 (41.9)	166 (44.4)	28 (31.5)	​
Female	269 (58.1)	208 (55.6)	61 (68.5)	​
Age (y), mean ± SD	70.9 ± 6.8	69.7 ± 6.2	75.9 ± 6.8	<0.001
Living status, n (%)	​	​	​	0.889
Living with others	409 (88.3)	330 (88.2)	79 (88.8)	​
Living alone	54 (11.7)	44 (11.8)	10 (11.2)	​
Hypertension, n (%)	​	​	​	0.006
No	321 (69.3)	270 (72.2)	51 (57.3)	​
Yes	142 (30.7)	104 (27.8)	38 (42.7)	​
DM, n (%)	​	​	​	0.287
No	283 (61.1)	233 (62.3)	50 (56.2)	​
Yes	180 (38.9)	141 (37.7)	39 (43.8)	​
Hyperlipidemia, n (%)	​	​	​	0.869
No	300 (64.8)	243 (65)	57 (64)	​
Yes	163 (35.2)	131 (35)	32 (36)	​
Smoking, n (%)	​	​	​	0.529
No	283 (61.1)	226 (60.4)	57 (64)	​
Yes	180 (38.9)	148 (39.6)	32 (36)	​
Alcohol, n (%)	​	​	​	0.6
No	317 (68.5)	254 (67.9)	63 (70.8)	​
Yes	146 (31.5)	120 (32.1)	26 (29.2)	​
BMI, mean ± SD	24.0 ± 3.2	24.2 ± 3.2	23.2 ± 3.4	0.006
TP (g/L), mean ± SD	68.0 ± 5.2	68.4 ± 5.1	66.6 ± 5.4	0.004
Alb (g/L), mean ± SD	40.0 ± 3.2	40.4 ± 3.2	38.3 ± 3.0	<0.001
BUN (mmol/L), mean ± SD	6.2 ± 1.5	6.3 ± 1.5	6.2 ± 1.4	0.582
Cr (μmol/L), mean ± SD	70.6 ± 15.9	70.9 ± 16.0	69.2 ± 15.4	0.36
CRP (mg/L), median (Q1, Q3)	7.8 (5.1, 12.6)	7.3 (5.0, 12.0)	10.2 (5.5, 17.4)	<0.001
WBC (10^9^/L), mean ± SD	6.3 ± 1.5	6.4 ± 1.6	6.2 ± 1.4	0.373
NEUT (10^9^/L), mean ± SD	3.6 ± 0.9	3.7 ± 0.9	3.5 ± 0.8	0.121
PLT (10^9^/L), mean ± SD	202.4 ± 47.6	201.1 ± 46.4	207.7 ± 52.3	0.245
LYM (10^9^/L), mean ± SD	1.8 ± 0.5	1.8 ± 0.5	1.7 ± 0.5	0.772
Hb (g/L), mean ± SD	139.7 ± 17.7	140.0 ± 18.3	138.7 ± 15.1	0.549
Psoas CSA (cm^2^), mean ± SD	4.0 ± 1.2	4.2 ± 1.1	2.9 ± 0.8	<0.001
Erector CSA (cm^2^), mean ± SD	7.8 ± 1.8	8.3 ± 1.6	5.6 ± 1.1	<0.001
Multifidus CSA (cm^2^), mean ± SD	3.1 ± 0.7	3.3 ± 0.6	2.3 ± 0.4	<0.001
Psoas fat (%), mean ± SD	13.3 ± 3.4	12.1 ± 2.1	18.5 ± 3.2	<0.001
Erector fat (%), mean ± SD	19.5 ± 4.8	17.7 ± 3.0	26.8 ± 4.2	<0.001
Multifidus fat (%), mean ± SD	24.8 ± 5.3	23.1 ± 3.9	32.4 ± 3.5	<0.001
Length of hospital stay (days), mean ± SD	8.7 ± 1.1	8.5 ± 1.0	9.5 ± 1.1	<0.001
Total CSA (cm^2^), mean ± SD	14.8 ± 3.5	15.8 ± 3.0	10.8 ± 2.1	<0.001
Mean fat (%), mean ± SD	19.2 ± 4.0	17.6 ± 2.2	25.9 ± 2.5	<0.001
Admission VAS, mean ± SD	5.8 ± 0.8	5.8 ± 0.8	5.9 ± 0.8	0.09
Discharge VAS, mean ± SD	3.4 ± 0.9	3.4 ± 0.9	3.7 ± 0.9	<0.001
Admission JOA, mean ± SD	10.3 ± 1.2	10.4 ± 1.2	9.8 ± 1.3	<0.001
Discharge JOA, mean ± SD	20.1 ± 0.8	20.2 ± 0.8	19.6 ± 0.9	<0.001
1-month JOA, mean ± SD	20.3 ± 1.9	20.8 ± 1.6	17.9 ± 1.3	<0.001
3-month JOA, mean ± SD	20.5 ± 2.3	21.1 ± 2.0	17.9 ± 1.6	<0.001
ASMI (kg/m^2^), mean ± SD	6.5 ± 1.4	6.8 ± 1.3	5.4 ± 1.0	<0.001
Gait speed (m/s), mean ± SD	27.3 ± 8.4	29.0 ± 8.2	20.6 ± 5.7	<0.001
Grip strength (kg), mean ± SD	1.1 ± 0.2	1.1 ± 0.1	1.0 ± 0.1	<0.001
Main stenosis level, n (%)	​	​	​	0.849
L2/3	7 (1.5)	6 (1.6)	1 (1.1)	​
L3/4	51 (11.0)	43 (11.5)	8 (9)	​
L4/5	349 (75.4)	278 (74.3)	71 (79.8)	​
L5/S1	56 (12.1)	47 (12.6)	9 (10.1)	​
Schizas grade, n (%)	​	​	​	0.441
Grade B	172 (37.1)	144 (38.5)	28 (31.5)	​
Grade C	205 (44.3)	161 (43)	44 (49.4)	​
Grade D	86 (18.6)	69 (18.4)	17 (19.1)	​
Analgesic use, n (%)	​	​	​	0.142
No	150 (32.4)	127 (34)	23 (25.8)	​
Yes	313 (67.6)	247 (66)	66 (74.2)	​
Analgesic type, n (%)	​	​	​	0.007
Acetaminophen	12 (2.6)	8 (2.1)	4 (4.5)	​
Gabapentin	10 (2.2)	4 (1.1)	6 (6.7)	​
Pregabalin	27 (5.8)	20 (5.3)	7 (7.9)	​
Celecoxib	264 (57.0)	215 (57.5)	49 (55.1)	​
None	150 (32.4)	127 (34)	23 (25.8)	​

Continuous variables conforming to normal distribution are expressed as the mean ± standard deviation (SD); skewed variable CRP is presented as median (interquartile range, Q1 and Q3); categorical variables are reported as count (%). Sarcopenia was diagnosed according to the 2019 Asian Working Group for Sarcopenia (AWGS) criteria.

Abbreviations: DM, diabetes mellitus; BMI, body mass index; TP, total protein; Alb, albumin; BUN, blood urea nitrogen; Cr, creatinine; CRP, C-reactive protein; WBC, white blood cell count; NEUT, neutrophil count; PLT, platelet count; LYM, lymphocyte count; Hb, hemoglobin; CSA, cross-sectional area; VAS, visual analog scale; JOA, Japanese Orthopedic Association score (0–29, higher scores denote superior function); ASMI, appendicular skeletal muscle mass index; gait speed, 6-meter walking velocity; grip strength, dominant hand grip force.

Psoas, erector spinae, and multifidus CSA and corresponding fat infiltration percentages were measured at the stenotic intervertebral disc level. Total paraspinal CSA equals the sum of the three individual muscle CSAs, and mean fat percentage is the average fat proportion of the three muscles.

Regarding paraspinal muscle parameters, the sarcopenia group had significantly smaller CSA of the psoas, erector spinae, and multifidus muscles and significantly higher fat percentages in all three muscle groups. Total paraspinal muscle CSA was also significantly smaller, and mean paraspinal muscle fat percentage was significantly higher in the sarcopenia group. The hospital stay was significantly longer in the sarcopenia group. Discharge VAS score was slightly higher than that in the non-sarcopenia group, and JOA scores at all follow-up time points were lower than those in the non-sarcopenia group.

Interobserver reliability for all measurements was good to excellent, with ICC values of 0.829 for psoas CSA, 0.896 for erector spinae CSA, 0.821 for multifidus CSA, 0.846 for psoas fat infiltration, 0.844 for erector spinae fat infiltration, 0.763 for multifidus fat infiltration, and 0.911 for Schizas grade (Supplementary Table S4).

### 3.3 Sarcopenia as an independent risk factor for discharge outcome

**FIGURE 1:**
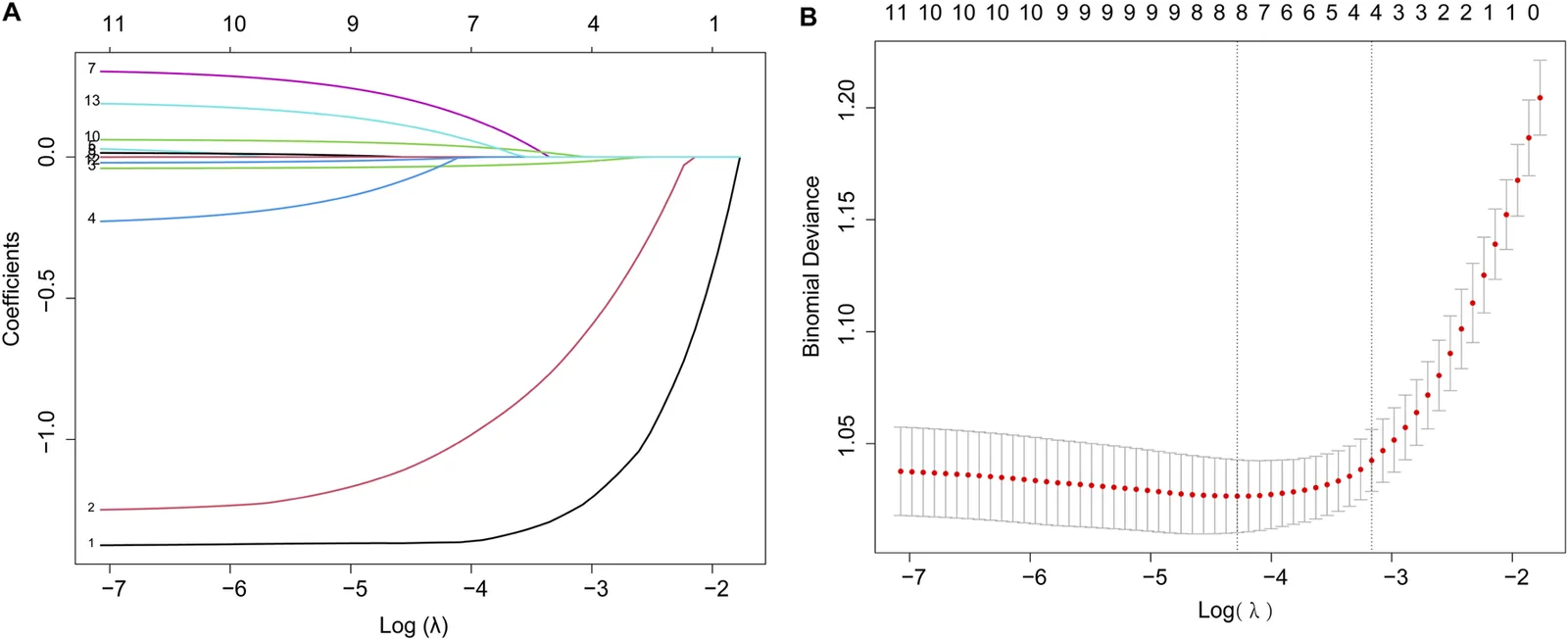
LASSO regression for variable selection. **(A)** Coefficient paths for each candidate predictor (assessed at baseline) across the penalty parameter λ. **(B)** Ten-fold cross-validation curve for optimal λ selection (1-SE rule). The outcome variable was good discharge outcome, defined as >50% improvement in the JOA score from admission to discharge.

Univariate logistic regression identified 12 variables with *p* < 0.1 for association with discharge outcome: sarcopenia, sex, age, hypertension, smoking, drinking, BMI, total protein, albumin, creatinine, CRP, and neutrophil count (Supplementary Table S5). Paraspinal muscle parameters and individual sarcopenia components (ASMI, gait speed, and grip strength) were not included in the variable selection process due to their strong collinearity with the binary sarcopenia variable and conceptual overlap. These 12 candidate variables were entered into LASSO regression. Using 10-fold cross-validation and the 1-SE rule, four variables had non-zero coefficients: sarcopenia, sex, age, and albumin. The LASSO variable selection process is shown in Figure 1.

Multicollinearity among the final predictors was assessed using variance inflation factors (VIFs); there was no multicollinearity among the variables (Supplementary Table S6). To assess the stability of our variable selection and to ensure that potentially important predictors were not overlooked, we performed a sensitivity analysis by directly entering all candidate variables into the LASSO regression; the same four predictors were retained, namely, sarcopenia, sex, age, and albumin (Supplementary Figures S1, S2).

A multivariable logistic regression model based on these four variables showed that after adjusting for sex, age, and albumin, sarcopenia remained an independent risk factor for poor outcome at discharge: odds ratio (OR) = 0.21 (95% confidence interval [CI]: 0.12–0.38, *p* < 0.001) (Table 2).

**TABLE 2:** Multivariable logistic regression of sarcopenia and discharge outcome.

Model	OR (95% CI)	*p*-value
Non-sarcopenia	1 (Reference)	​
Unadjusted	0.15 (0.09∼0.24)	<0.001
Adjusted for sex	0.15 (0.09∼0.25)	<0.001
Adjusted for sex and age	0.2 (0.11∼0.34)	<0.001
Adjusted for sex, age, and albumin	0.21 (0.12∼0.38)	<0.001

Abbreviations: OR, odds ratio; CI, confidence intervals.

### 3.4 Mediation analysis of paraspinal muscle parameters

**FIGURE 2:**
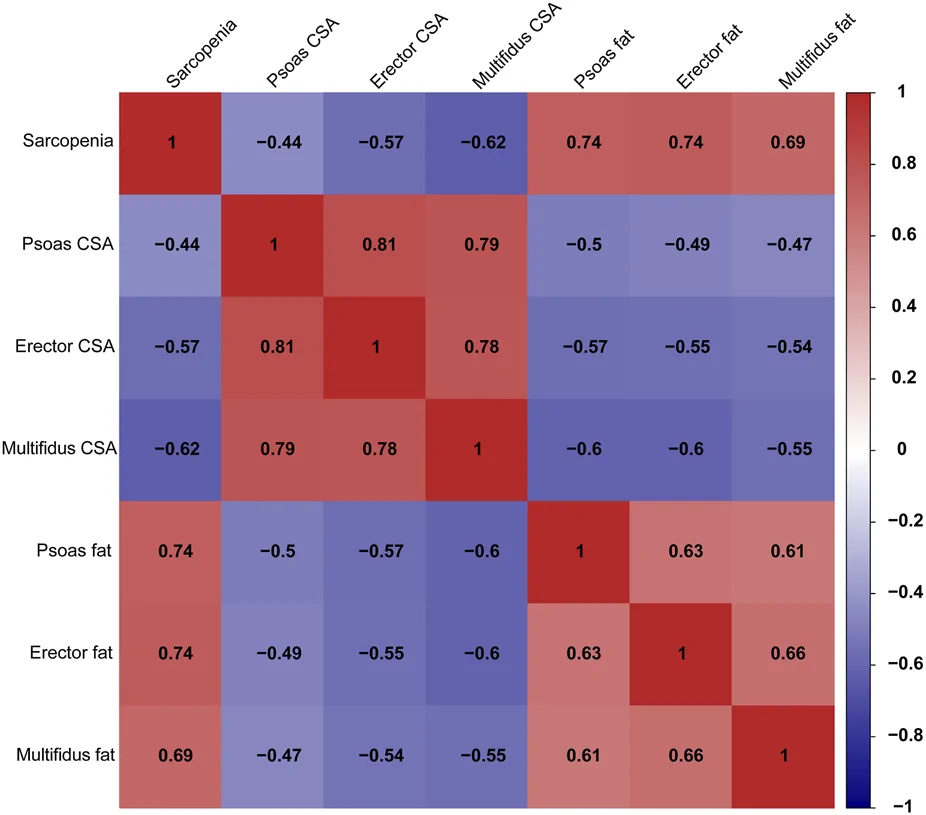
Correlation matrix of baseline sarcopenia and paraspinal muscle parameters. Pairwise Pearson correlations between sarcopenia (AWGS-defined) and paraspinal CSA and fat percentage (psoas, erector spinae, multifidus, total CSA, and mean fat percentage), all measured at baseline. Color intensity indicates the magnitude of the correlation; blue and red denote negative and positive correlations, respectively.

Given the strong collinearity between sarcopenia and paraspinal muscle parameters, as well as among the parameters themselves (Figure 2), total paraspinal muscle CSA and mean paraspinal muscle fat percentage were analyzed as separate mediators. The bootstrap method with 5,000 resamples was used to estimate mediation effects.

The mediation analysis showed that the total paraspinal muscle CSA had a significant mediation effect: ACME = −0.23 (95% CI: −0.37 to −0.06, p = 0.012); proportion mediated = 74.64% (Table 3). The mean paraspinal muscle fat percentage showed no significant mediation effect (*p* = 0.71), indicating that sarcopenia influences treatment outcomes mainly through reduced paraspinal muscle CSA rather than increased fat infiltration.

**TABLE 3:** Mediation analysis of paraspinal muscle parameters on the association between sarcopenia and discharge outcome.

Mediator	Effect	Estimate (95% CI)	*p*-value
Total CSA	ACME	−0.23 (−0.37 to −0.06)	0.012
	ADE	−0.08 (−0.30 to 0.11)	0.464
	Total effect	−0.30 (−0.42 to −0.18)	<0.001
	Proportion (%)	74.64	0.012
Mean fat percentage	ACME	−0.03 (−0.20 to 0.11)	0.710
	ADE	−0.28 (−0.49 to −0.07)	0.012
	Total effect	−0.31 (−0.44 to −0.19)	<0.001
	Proportion (%)	10.10	0.710

Mediation analyses were performed using the bootstrap method with 5,000 resamples. ACME, average causal mediation effect; ADE, average direct effect; CSA, cross-sectional area. Proportion mediated is expressed as the percentage of the total effect mediated.

### 3.5 Sensitivity analyses

We performed a series of sensitivity analyses to assess the robustness of our primary findings against potential biases, including missing data, model specification, sparse data bias, and subgroup heterogeneity (Supplementary Tables S7–S9).

First, to evaluate the impact of missing data on our primary findings, we performed a complete case analysis, excluding patients with any missing data. Sarcopenia remained significantly associated with poor discharge outcome (OR = 0.21, 95% CI: 0.12–0.37, *p* < 0.001), consistent with the primary analysis (Supplementary Table S7). In addition, using length of hospital stay as a continuous outcome, multivariable linear regression adjusted for age, sex, and albumin showed that sarcopenia was associated with a significantly prolonged hospital stay (β = 1.01 days, 95% CI: 0.76–1.26, *p* < 0.001) (Supplementary Table S7).

Second, given the concern regarding sparse data bias in the 1-month and 3-month analyses, where only 8 and 14 sarcopenic patients achieved good outcomes, respectively, we applied Firth’s penalized maximum likelihood estimation to reduce potential bias in logistic regression with rare events (Heinze and Schemper, 2002). In multivariable models adjusting for sex, age, and albumin, the Firth-corrected ORs were 0.022 (95% CI: 0.008–0.054) for the 1-month outcome and 0.058 (95% CI: 0.027–0.117) for the 3-month outcome. In simplified models adjusting for individual confounders (age, sex, or albumin, separately), the Firth-corrected ORs ranged from 0.015 to 0.045 for 1-month outcomes and from 0.040 to 0.079 for 3-month outcomes (Supplementary Table S8). All estimates remained statistically significant (all *p* < 0.001), and the direction of effect was consistent with the primary analysis, supporting the robustness of our main findings.

**FIGURE 3:**
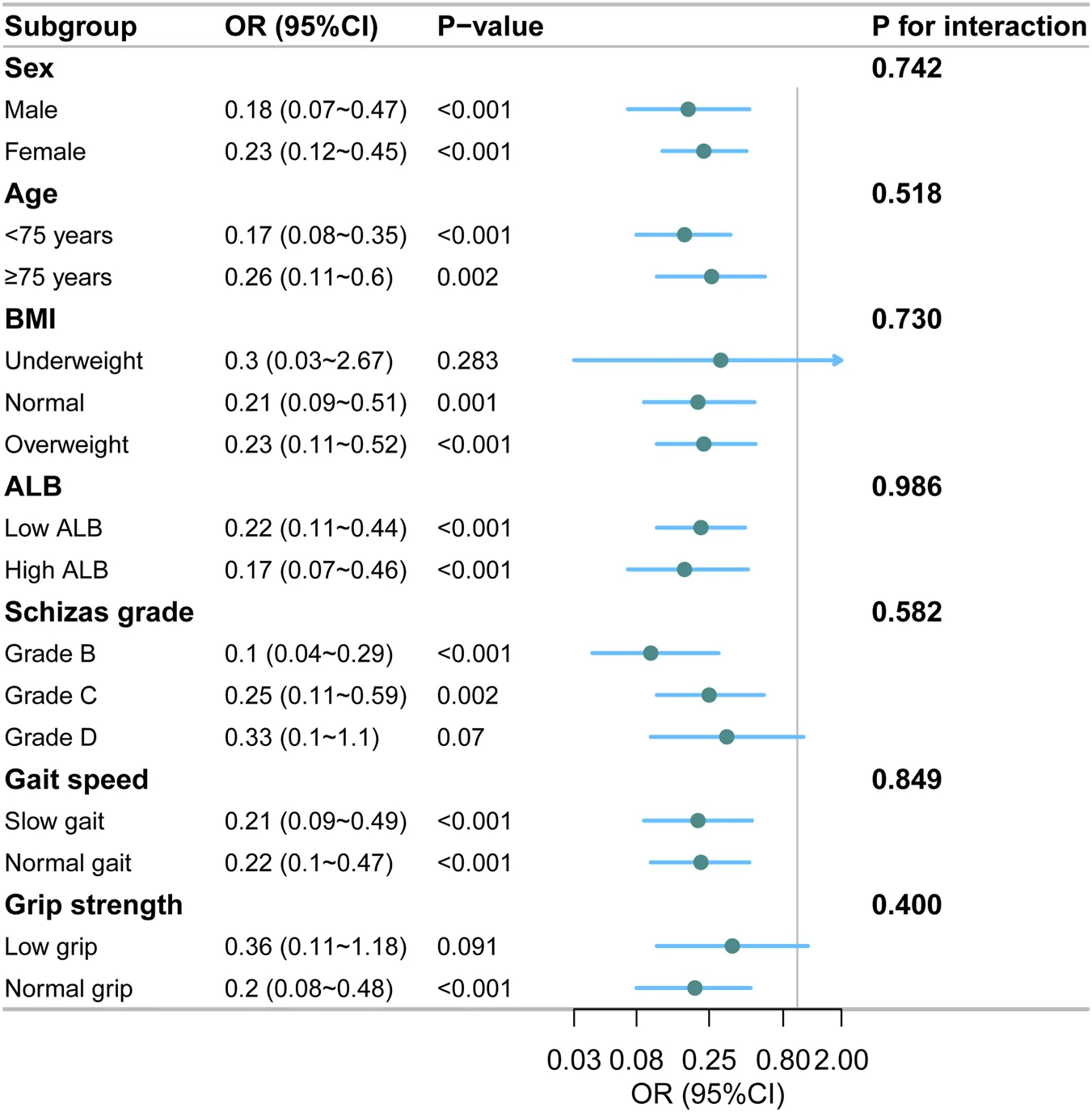
Forest plot of subgroup analyses for the association between baseline sarcopenia and poor discharge outcome. Adjusted odds ratios and 95% confidence intervals for the association between sarcopenia and poor discharge outcome, stratified by sex, age (<75 and ≥75 years), BMI (underweight <18.5, normal 18.5–23.9, and overweight ≥24.0 kg/m^2^), albumin (stratified by median value: <40 and ≥40 g/L), Schizas grade (B, C, and D), gait speed (≤1 and >1 m/s), and grip strength (low vs. normal, using sex-specific AWGS 2019 cutoffs). All estimates were adjusted for age, sex, and albumin, except for the stratification variable.

Third, subgroup analyses stratified by sex, age, BMI, albumin, Schizas grade, gait speed, and grip strength showed that the detrimental effect of sarcopenia remained consistent across most subgroups, with no significant interactions (all *p* for interaction >0.05) (Figure 3). These findings support the generalizability of our results across different patient strata.

Fourth, we examined the independent associations of sarcopenia components with discharge outcome (Supplementary Table S9). In univariate analyses, all three components were significantly associated with poor outcome. After adjusting for age, sex, and albumin, ASMI (adjusted OR = 2.01, 95% CI: 1.36–2.97, *p* < 0.001) and grip strength (adjusted OR = 1.13, 95% CI: 1.06–1.20, *p* < 0.001) remained independent predictors, whereas gait speed was no longer significant (adjusted OR = 1.45, 95% CI: 0.25–8.20, *p* = 0.677). These findings suggest that muscle mass and strength, rather than physical performance, may be more directly associated with short-term outcomes in this population.

## 4 Discussion

This prospective cohort study systematically evaluated the impact of sarcopenia on short-term outcomes of conservative treatment in elderly LSS patients. The prevalence of sarcopenia was 19.2%, and sarcopenia was associated with a significantly higher risk of poor discharge outcome (OR = 0.21 for good outcome, equivalent to approximately 5-fold higher odds of poor outcome). Mediation analysis further revealed that this effect was primarily driven by reduced paraspinal muscle CSA, which accounted for 74.64% of the total effect.

The 19.2% prevalence in our cohort aligns closely with the 19.7% reported by Matsuo et al. (2020) in a Japanese LSS population and is nearly double the estimated 10% prevalence in community-dwelling older adults (Xie et al., 2026), suggesting that LSS is closely linked to systemic aging-related syndromes beyond local spinal degeneration. Patients with sarcopenia also exhibited significantly higher CRP and lower albumin levels. The elevated CRP is consistent with the inflammatory–catabolic axis of sarcopenia (Pan et al., 2021; Shokri-Mashhadi et al., 2021), while reduced albumin reflects the nutritional dimension of muscle loss (Barone et al., 2025). The higher prevalence of hypertension in the sarcopenia group further supports shared pathophysiological mechanisms involving oxidative stress and chronic inflammation (Bai et al., 2020; Quan et al., 2023; Zou et al., 2024), implying that sarcopenia screening should extend beyond the musculoskeletal system into a broader multi-system framework.

Multivariable logistic regression showed that sarcopenia remained an independent risk factor for poor discharge outcomes after adjusting for sex, age, and albumin, consistent with surgical cohort evidence that sarcopenia impairs postoperative functional recovery (Chen et al., 2024a). Jin and Wi (2025) previously found that paraspinal muscle degeneration predicts conservative treatment failure, and our mediation analysis further quantifies this causal chain. Stratified analysis of AWGS sarcopenia components revealed that ASMI and grip strength independently predicted poor outcomes, while gait speed lost statistical significance. This aligns with prior spine research (Sakai et al., 2020), yet contradicts Stache et al. (2026), who identified gait speed as the primary pain predictor in heterogeneous chronic back pain populations. The discrepancy may be partly explained by differences in study populations and outcome measures. Stache et al. (2026) examined a mixed musculoskeletal pain cohort in a cross-sectional design, where gait speed emerged as a broad indicator of pain severity across heterogeneous etiologies. In contrast, our longitudinal study focused on functional recovery in a homogeneous LSS population, where gait impairment is likely driven more by neurogenic claudication than by musculoskeletal decline. In this specific context, muscle mass and strength appear to better predict functional recovery after conservative treatment. Additionally, the markedly lower ORs observed at 1-month and 3-month follow-up may be attributable to sparse data bias as only 8 and 14 sarcopenic patients achieved satisfactory recovery, respectively. After applying Firth’s penalized regression correction, all medium-term associations remained statistically significant, confirming that sarcopenia exerts a sustained adverse effect, despite the instability of the effect estimates.

Mediation analysis indicated that total paraspinal CSA mediated 74.64% of the sarcopenia-outcome association, while mean fat infiltration had no mediating effect. Due to the inherent risk of unmeasured confounding in observational mediation models and the fact that mediation analysis itself cannot establish causality, this statistical association should not be interpreted as definitive proof of a causal biological pathway (Richiardi et al., 2013; VanderWeele, 2016). Conflicting literature exists on fat infiltration’s prognostic value: Lawan et al. (2025) reported that fat infiltration correlates with LSS diagnosis, but not pain or disability; Jin and Wi (2025) found that lower paraspinal CSA/BMI, rather than fatty infiltration, independently predicted conservative treatment failure. Our mediation analysis offers a potential explanation for this discrepancy by decomposing the pathway: reduced muscle mass, not fat infiltration, mediated the sarcopenia–outcome association, suggesting that fat infiltration may be a bystander marker of chronic degeneration, rather than a direct driver of poor rehabilitation. Biomechanically, smaller paraspinal CSA weakens lumbar dynamic stabilization and aggravates neural compression (Stevens et al., 2020; Nakamura et al., 2022; Chen et al., 2024b), whereas intramuscular fat does not impair muscle contractile function, explaining its negligible mediating role. Notably, paraspinal CSA did not fully mediate the sarcopenia–outcome association, suggesting that other pathways may also be involved. Future studies are needed to further elucidate these potential mechanisms.

From a clinical perspective, our findings support sarcopenia as a useful risk stratification tool. The detrimental effect remained consistent across diverse patient subgroups, supporting its generalizability. Patients with sarcopenia can be informed of their approximately 5-fold higher odds of poor outcomes, facilitating realistic expectation setting and closer monitoring during conservative treatment. However, sarcopenia status should not be used as a sole indication for earlier surgical referral; rather, it should be considered alongside symptom severity, functional impairment, and patient preferences. Although we cannot recommend specific interventions such as nutritional support or resistance training from our data, these findings generate hypotheses for future interventional studies. Incorporating paraspinal CSA measurements into routine LSS provides additional prognostic information at no extra burden. However, it is not a substitute for multidimensional sarcopenia evaluation, particularly grip strength and ASMI. Instead, MRI parameters should be interpreted together with these clinical measures.

The limitations of this study also warrant attention. First, paraspinal muscle parameters were measured at a single lumbar level rather than across multiple levels. Although this approach is common in LSS research, muscle CSA and fatty infiltration may vary considerably across lumbar levels (Sasaki et al., 2017; Berry et al., 2018; Urrutia et al., 2018). The use of a single-level measurement may, therefore, not fully capture the overall morphological characteristics of the paraspinal muscles. Second, the conservative treatment protocol consisted of multiple combined interventions, making it difficult to determine which specific component contributed most to clinical improvement. Future factorial randomized controlled trials are needed to clarify the effectiveness of each individual measure. Third, the analgesic component was not completely uniform. Patients with sarcopenia had higher rates of analgesic use and greater reliance on gabapentinoids than non-sarcopenic patients. Despite receiving more intensive analgesic regimens, sarcopenic patients still exhibited significantly worse clinical outcomes, and recent evidence suggests that gabapentinoids have limited additional efficacy in LSS (Martínez et al., 2023; Chotigavanichaya et al., 2025). Thus, the observed detrimental effect of sarcopenia is unlikely to be explained by analgesic heterogeneity. Fourth, the primary outcome was assessed at discharge, representing a short-term rather than long-term response. The natural history of conservatively treated LSS is highly variable (Minamide et al., 2013; Tsubosaka et al., 2018), with pain and disability improvements occurring primarily within the first year (Mc Auliffe et al., 2026). The high proportion of missing 3-month JOA scores (29.8%) precluded longer-term analysis, and future large-sample, long-term follow-up studies are warranted. Fifth, this was a single-center study, which may limit generalizability; validation in multi-center cohorts is needed.

## 5 Conclusion

Sarcopenia is an independent risk factor for poor short-term outcomes after conservative treatment in elderly LSS patients, an effect primarily mediated by reduced paraspinal muscle CSA. These findings support the use of sarcopenia status in risk stratification and shared decision-making. However, whether targeted interventions for sarcopenia can improve conservative treatment outcomes requires further investigation.

## Data Availability

The raw data supporting the conclusions of this article will be made available by the authors, without undue reservation.

## References

[B1] Alicioglu B. Yilmaz B. Bulakbasi N. Copuroglu C. Yalniz E. Aykac B. (2012). Magnetic resonance imaging predictors of surgical outcome in degenerative lumbar spinal stenosis. Jpn. J. Radiol. 30, 811–818. 10.1007/s11604-012-0125-0 22968746

[B2] Ammendolia C. Hofkirchner C. Plener J. Bussières A. Schneider M. J. Young J. J. (2022). Non-operative treatment for lumbar spinal stenosis with neurogenic claudication: an updated systematic review. BMJ Open 12, e057724. 10.1136/bmjopen-2021-057724 35046008 PMC8772406

[B3] Bagley C. MacAllister M. Dosselman L. Moreno J. Aoun S. G. El Ahmadieh T. Y. (2019). Current concepts and recent advances in understanding and managing lumbar spine stenosis. F1000Res 8, 137. 10.12688/f1000research.16082.1 30774933 PMC6357993

[B4] Bai T. Fang F. Li F. Ren Y. Hu J. Cao J. (2020). Sarcopenia is associated with hypertension in older adults: a systematic review and meta-analysis. BMC Geriatr. 20, 279. 10.1186/s12877-020-01672-y 32762638 PMC7409686

[B5] Barone M. Baccaro P. Molfino A. (2025). An overview of sarcopenia: focusing on nutritional treatment approaches. Nutrients 17, 1237. 10.3390/nu17071237 40218995 PMC11990658

[B6] Berry D. B. Padwal J. Johnson S. Parra C. L. Ward S. R. Shahidi B. (2018). Methodological considerations in region of interest definitions for paraspinal muscles in axial MRIs of the lumbar spine. BMC Musculoskelet. Disord. 19, 135. 10.1186/s12891-018-2059-x 29734942 PMC5938809

[B7] Chatterjee S. Hastie T. Tibshirani R. (2025). Univariate-guided sparse regression. Harv. Data Sci. Rev. 7 (3). 10.1162/99608f92.c79ff6db PMC13505989 42644161

[B8] Chen L.-K. Woo J. Assantachai P. Auyeung T.-W. Chou M.-Y. Iijima K. (2020). Asian working group for Sarcopenia: 2019 consensus update on sarcopenia diagnosis and treatment. J. Am. Med. Dir. Assoc. 21, 300–307.e2. 10.1016/j.jamda.2019.12.012 32033882

[B9] Chen M. J.-W. Lo Y.-S. Lin C.-Y. Tseng C. Hsiao P.-H. Lai C.-Y. (2024a). Impact of sarcopenia on outcomes following lumbar spine surgery for degenerative disease: an updated systematic review and meta-analysis. Eur. Spine J. 33, 3369–3380. 10.1007/s00586-024-08364-w 38907066

[B10] Chen P. Zhou Z. Sun L. Yu X. Li K. Li J. (2024b). Quantitative multi-parameter assessment of age- and gender-related variation of back extensor muscles in healthy adults using Dixon MR imaging. Eur. Radiol. 34, 69–79. 10.1007/s00330-023-09954-w 37537425

[B11] Chotigavanichaya C. Mekariya K. Santipas B. Wilartratsami S. Korwutthikulrangsri E. Ruangchainikom M. (2025). Efficacy of gabapentin and pregabalin for the treatment of neurogenic claudication in lumbar spinal stenosis: a double-blind randomized placebo-controlled trial. Asian Spine J. 19, 916–927. 10.31616/asj.2025.0096 40858318 PMC12765924

[B12] Chua M. Hochberg U. Regev G. Ophir D. Salame K. Lidar Z. (2022). Gender differences in multifidus fatty infiltration, sarcopenia and association with preoperative pain and functional disability in patients with lumbar spinal stenosis. Spine J. 22, 58–63. 10.1016/j.spinee.2021.06.007 34111552

[B13] Davison M. A. Lilly D. T. Moreno J. Bagley C. Adogwa O. (2021). A comparison of successful *versus* failed nonoperative treatment approaches in patients with degenerative conditions of the lumbar spine. J. Clin. Neurosci. 86, 71–78. 10.1016/j.jocn.2020.12.033 33775350

[B14] Deer T. Sayed D. Michels J. Josephson Y. Li S. Calodney A. K. (2019). A review of lumbar spinal stenosis with intermittent neurogenic claudication: disease and diagnosis. Pain Med. 20, S32–S44. 10.1093/pm/pnz161 31808530 PMC7101166

[B15] Ekşi M. Ş. Topçu A. Topaloğlu F. Tanriverdi N. Yeşilyurt S. C. Duymaz U. C. (2025). Fatty infiltration in the multifidus predicts screw-loosening following short-segment decompression and fusion: proof of why we should protect and rehabilitate the paraspinal muscles. Eur. Spine J. 34, 2427–2437. 10.1007/s00586-025-08793-1 40140014

[B16] Heinze G. Schemper M. (2002). A solution to the problem of separation in logistic regression. Stat. Med. 21, 2409–2419. 10.1002/sim.1047 12210625

[B17] Jensen R. K. Jensen T. S. Koes B. Hartvigsen J. (2020). Prevalence of lumbar spinal stenosis in general and clinical populations: a systematic review and meta-analysis. Eur. Spine J. 29, 2143–2163. 10.1007/s00586-020-06339-1 32095908

[B18] Jensen R. K. Harhangi B. S. Huygen F. Koes B. (2021). Lumbar spinal stenosis. BMJ 373, n1581. 10.1136/bmj.n1581 34187838

[B19] Jin J. Wi S. M. (2025). The impact of paraspinal sarcopenia compared with generalized sarcopenia on conservative treatment outcomes in degenerative lumbar spinal stenosis. Clin. Spine Surg. 10.1097/BSD.0000000000001945 41186986

[B20] Katz J. N. Zimmerman Z. E. Mass H. Makhni M. C. (2022). Diagnosis and management of lumbar spinal stenosis: a review. JAMA 327, 1688–1699. 10.1001/jama.2022.5921 35503342

[B21] Kim H.-Y. (2013). Statistical notes for clinical researchers: assessing normal distribution (2) using skewness and kurtosis. Restor. Dent. Endod. 38, 52–54. 10.5395/rde.2013.38.1.52 23495371 PMC3591587

[B22] Koo T. K. Li M. Y. (2016). A guideline of selecting and reporting intraclass correlation coefficients for reliability research. J. Chiropr. Med. 15, 155–163. 10.1016/j.jcm.2016.02.012 27330520 PMC4913118

[B23] Kreiner D. S. Shaffer W. O. Baisden J. L. Gilbert T. J. Summers J. T. Toton J. F. (2013). An evidence-based clinical guideline for the diagnosis and treatment of degenerative lumbar spinal stenosis (update). Spine J. 13, 734–743. 10.1016/j.spinee.2012.11.059 23830297

[B24] Lawan A. Crites Videman Z. Belay A. Behery S. Ibrahim S. Ulrich T. (2025). Are paraspinal muscle morphology and composition associated with lumbar spinal stenosis? A systematic review. Spine J. 25, 1794–1820. 10.1016/j.spinee.2025.01.027 39922502

[B25] Martínez T. Mariscal G. de la Rubia Ortí J. E. Barrios C. (2023). Efficacy and safety of pregabalin and gabapentin in spinal stenosis: a systematic review and meta-analysis. Front. Pharmacol. 14, 1249478. 10.3389/fphar.2023.1249478 38094885 PMC10716263

[B26] Matsuo S. Kawakami M. Minetama M. Nakagawa M. Teraguchi M. Kagotani R. (2020). Clinical features of sarcopenia in patients with lumbar spinal stenosis. Spine (Phila Pa 1976) 45, E1105–E1110. 10.1097/BRS.0000000000003498 32205696

[B27] Mc Auliffe S. Kirby E. Mockler D. Farooq A. Trine Fogh Johansson S. Krüger Jensen R. (2026). The long-term clinical course of non-surgically treated lumbar spinal stenosis: a systematic review and meta-analysis. Eur. Spine J. 35, 452–470. 10.1007/s00586-025-09502-8 41148274

[B28] Minamide A. Yoshida M. Maio K. (2013). The natural clinical course of lumbar spinal stenosis: a longitudinal cohort study over a minimum of 10 years. J. Orthop. Sci. 18, 693–698. 10.1007/s00776-013-0435-9 23839003

[B29] Minetama M. Kawakami M. Teraguchi M. Nakagawa M. Yamamoto Y. Sakon N. (2024). Minimal clinically important differences in walking capacity and physical activity after nonsurgical treatment in patients with lumbar spinal stenosis: a secondary analysis of a randomized controlled trial. Spine J. 24, 256–262. 10.1016/j.spinee.2023.10.011 37871657

[B30] Naidu R. K. Tran O. V. Schatman M. E. (2024). Longitudinal analysis of the care pathway of patients with lumbar spinal stenosis in the US. J. Pain Res. 17, 1979–1987. 10.2147/JPR.S454887 38854929 PMC11162185

[B31] Nakamura M. Otani K. Kaneko Y. Sekiguchi M. Konno S.-I. (2022). The relationship between exercise-induced low back pain, the fat infiltration rate of paraspinal muscles, and lumbar sagittal balance. Spine Surg. Relat. Res. 6, 261–270. 10.22603/ssrr.2021-0103 35800627 PMC9200416

[B32] Narayanan R. Ezeonu T. Kellish A. Somers S. Lee Y. Khanna A. (2025). Does paraspinal muscle mass predict lumbar lordosis before and after decompression for degenerative spinal stenosis? Spine (Phila Pa 1976) 50, E29–E35. 10.1097/BRS.0000000000005058 38809843

[B33] Pan L. Xie W. Fu X. Lu W. Jin H. Lai J. (2021). Inflammation and sarcopenia: a focus on circulating inflammatory cytokines. Exp. Gerontol. 154, 111544. 10.1016/j.exger.2021.111544 34478826

[B34] Quan Y. Wang C. Wang L. Li G. (2023). Geriatric sarcopenia is associated with hypertension: a systematic review and meta-analysis. J. Clin. Hypertens. (Greenwich) 25, 808–816. 10.1111/jch.14714 37594142 PMC10497027

[B35] Regev G. J. Hochberg U. Ofir D. Salame K. Lidar Z. Khashan M. (2026). Impact of sarcopenia and multifidus atrophy on outcomes from minimally invasive decompression surgery for lumbar spinal stenosis. N. Am. Spine Soc. J. 25, 100828. 10.1016/j.xnsj.2025.100828 41503350 PMC12769823

[B36] Richiardi L. Bellocco R. Zugna D. (2013). Mediation analysis in epidemiology: methods, interpretation and bias. Int. J. Epidemiol. 42, 1511–1519. 10.1093/ije/dyt127 24019424

[B37] Sakai Y. Wakao N. Matsui H. Tomita K. Watanabe T. Iida H. (2020). Surgical results in older patients with lumbar spinal stenosis according to gait speed in relation to the diagnosis for sarcopenia. J. Orthop. Surg. Hong. Kong 28, 2309499020918422. 10.1177/2309499020918422 32329390

[B38] Salo V. Määttä J. Takala J. Heikkilä A. Reimann E. (2026). Genetic architecture of lumbar spinal stenosis. Nat. Commun. 17, 4920. 10.1038/s41467-026-70935-w 41942452 PMC13234025

[B39] Sasaki T. Yoshimura N. Hashizume H. Yamada H. Oka H. Matsudaira K. (2017). MRI-defined paraspinal muscle morphology in Japanese population: the Wakayama spine study. PLoS One 12, e0187765. 10.1371/journal.pone.0187765 29117256 PMC5678698

[B40] Schizas C. Theumann N. Burn A. Tansey R. Wardlaw D. Smith F. W. (2010). Qualitative grading of severity of lumbar spinal stenosis based on the morphology of the dural sac on magnetic resonance images. Spine (Phila Pa 1976) 35, 1919–1924. 10.1097/BRS.0b013e3181d359bd 20671589

[B41] Schneider M. J. Ammendolia C. Murphy D. R. Glick R. M. Hile E. Tudorascu D. L. (2019). Comparative clinical effectiveness of nonsurgical treatment methods in patients with lumbar spinal stenosis: a randomized clinical trial. JAMA Netw. Open 2, e186828. 10.1001/jamanetworkopen.2018.6828 30646197 PMC6324321

[B42] Shipley J. A. (2008). Lumbar spinal stenosis. SA Orthop. J. 7, 42–49.

[B43] Shokri-Mashhadi N. Moradi S. Heidari Z. Saadat S. (2021). Association of circulating C-reactive protein and high-sensitivity C-reactive protein with components of sarcopenia: a systematic review and meta-analysis of observational studies. Exp. Gerontol. 150, 111330. 10.1016/j.exger.2021.111330 33848566

[B44] Stache S. Furey G. Mehallo C. J. (2026). The correlation between spatiotemporal gait and pain in patients with musculoskeletal pain using smartphone-based gait technology: a retrospective cross sectional study. BMC Musculoskelet. Disord. 10.1186/s12891-026-10203-6 42437907

[B45] Stevens S. Agten A. Timmermans A. Vandenabeele F. (2020). Unilateral changes of the multifidus in persons with lumbar disc herniation: a systematic review and meta-analysis. Spine J. 20, 1573–1585. 10.1016/j.spinee.2020.04.007 32325246

[B46] Tsubosaka M. Kaneyama S. Yano T. Kasahara K. Kanemura A. Takabatake M. (2018). The factors of deterioration in long-term clinical course of lumbar spinal canal stenosis after successful conservative treatment. J. Orthop. Surg. Res. 13, 239. 10.1186/s13018-018-0947-2 30227869 PMC6145329

[B47] Urrutia J. Besa P. Lobos D. Andia M. Arrieta C. Uribe S. (2018). Is a single-level measurement of paraspinal muscle fat infiltration and cross-sectional area representative of the entire lumbar spine? Skelet. Radiol. 47, 939–945. 10.1007/s00256-018-2902-z 29476224

[B48] VanderWeele T. J. (2016). Mediation analysis: a practitioner’s guide. Annu. Rev. Public Health 37, 17–32. 10.1146/annurev-publhealth-032315-021402 26653405

[B49] Webb C. W. Aguirre K. Seidenberg P. H. (2024). Lumbar spinal stenosis: diagnosis and management. Am. Fam. Physician 109, 350–359. 38648834

[B50] Xie C. Yan R. Tao R. (2026). Combined resistance training and amino acid-based supplementation for sarcopenia in older adults: a systematic review and meta-analysis. BMC Musculoskelet. Disord. 27, 35. 10.1186/s12891-025-09436-8 41540398 PMC12809905

[B51] Yuan S. Larsson S. C. (2023). Epidemiology of sarcopenia: prevalence, risk factors, and consequences. Metabolism 144, 155533. 10.1016/j.metabol.2023.155533 36907247

[B52] Zhu L. Sun Y. Kang J. Liang J. Su T. Fu W. (2024). Effect of acupuncture on neurogenic claudication among patients with degenerative lumbar spinal stenosis: a randomized clinical trial. Ann. Intern Med. 177, 1048–1057. 10.7326/M23-2749 38950397

[B53] Zou J. Liu Y. Tian C. Wang L. Li S. Ran J. (2024). Understanding the complexity of hypertension with Sarcopenia by scientometric analysis. J. Multidiscip. Healthc. 17, 6211–6228. 10.2147/JMDH.S498799 39759086 PMC11697656

