# Supplementary material for: Effect of sarcopenia and paraspinal muscle morphology on conservative treatment outcomes in lumbar spinal stenosis: a prospective cohort study
Source: Front Aging. 2026 Aug 26;7:1913710. doi: 10.3389/fragi.2026.1913710 (PMC13553900; doi:10.3389/fragi.2026.1913710)
Supplement: Supplementary file 1 [file Supplementaryfile1.docx]

Supplementary Figures:

Supplementary Figure 1. Coefficient paths in the full-variable LASSO regression.
