# Supplementary material for: Effect of sarcopenia and paraspinal muscle morphology on conservative treatment outcomes in lumbar spinal stenosis: a prospective cohort study
Source: Front Aging. 2026 Aug 26;7:1913710. doi: 10.3389/fragi.2026.1913710 (PMC13553900; doi:10.3389/fragi.2026.1913710)
Supplement: Supplementary file 2 [file Supplementaryfile2.docx]

Supplementary Figure 2. Ten-fold cross-validation curve for optimal λ selection in the full-variable LASSO sensitivity analysis. All candidate predictors (including Schizas grade and Analgesic use) were entered directly into the LASSO regression to assess the stability of variable selection. The optimal λ was selected using 10-fold cross-validation (1-SE rule). The four predictors (sarcopenia, sex, age, and albumin) were retained.
