# Supplementary material for: Effect of sarcopenia and paraspinal muscle morphology on conservative treatment outcomes in lumbar spinal stenosis: a prospective cohort study
Source: Front Aging. 2026 Aug 26;7:1913710. doi: 10.3389/fragi.2026.1913710 (PMC13553900; doi:10.3389/fragi.2026.1913710)
Supplement: Supplementary file 3 [file Table1.docx]

| **Supplementary Table 1. Comparison of descriptive statistics between unimputed and imputed data (n=463)** | | | | |
| --- | --- | --- | --- | --- |
| Variables | Missing cases, n (%) | Before imputation | After imputation | P value |
| Sarcopenia, n (%) | 0 (0.00%) |  |  | 1 |
| No |  | 374 (80.8) | 374 (80.8) |  |
| Yes |  | 89 (19.2) | 89 (19.2) |  |
| Sex, n (%) | 0 (0.00%) |  |  | 1 |
| Male |  | 194 (41.9) | 194 (41.9) |  |
| Female |  | 269 (58.1) | 269 (58.1) |  |
| Age (y), Mean ± SD | 0 (0.00%) | 70.9 ± 6.8 | 70.9 ± 6.8 | 1 |
| Living status, n (%) | 45 (9.72%) |  |  | 0.934 |
| Living with others |  | 370 (88.5) | 409 (88.3) |  |
| Living alone |  | 48 (11.5) | 54 (11.7) |  |
| Hypertension, n (%) | 20 (4.32%) |  |  | 0.992 |
| No |  | 307 (69.3) | 321 (69.3) |  |
| Yes |  | 136 (30.7) | 142 (30.7) |  |
| DM, n (%) | 16 (3.46%) |  |  | 0.638 |
| No |  | 280 (62.6) | 283 (61.1) |  |
| Yes |  | 167 (37.4) | 180 (38.9) |  |
| Hyperlipidemia, n (%) | 28 (6.05%) |  |  | 0.765 |
| No |  | 286 (65.7) | 300 (64.8) |  |
| Yes |  | 149 (34.3) | 163 (35.2) |  |
| Smoking, n (%) | 15 (3.24%) |  |  | 0.954 |
| No |  | 273 (60.9) | 283 (61.1) |  |
| Yes |  | 175 (39.1) | 180 (38.9) |  |
| Alcohol, n (%) | 35 (7.56%) |  |  | 0.938 |
| No |  | 292 (68.2) | 317 (68.5) |  |
| Yes |  | 136 (31.8) | 146 (31.5) |  |
| BMI, Mean ± SD | 12 (2.59%) | 24.1 ± 3.2 | 24.0 ± 3.2 | 0.865 |
| TP (g/L), Mean ± SD | 29 (6.26%) | 68.1 ± 5.2 | 68.0 ± 5.2 | 0.916 |
| Alb (g/L), Mean ± SD | 29 (6.26%) | 40.0 ± 3.2 | 40.0 ± 3.2 | 0.993 |
| BUN (mmol/L), Mean ± SD | 14 (3.02%) | 6.2 ± 1.5 | 6.2 ± 1.5 | 0.877 |
| Cr (μmol/L), Mean ± SD | 14 (3.02%) | 70.6 ± 16.1 | 70.6 ± 15.9 | 0.966 |
| CRP (mg/L), Median(Q1, Q3) | 6 (1.30%) | 7.8 (5.1, 12.6) | 7.8 (5.1, 12.6) | 0.984 |
| WBC (10^9/L), Mean ± SD | 19 (4.10%) | 6.3 ± 1.5 | 6.3 ± 1.5 | 0.922 |
| NEUT (10^9/L), Mean ± SD | 19 (4.10%) | 3.6 ± 0.9 | 3.6 ± 0.9 | 0.969 |
| PLT (10^9/L), Mean ± SD | 19 (4.10%) | 201.5 ± 48.0 | 202.4 ± 47.6 | 0.772 |
| LYM (10^9/L), Mean ± SD | 19 (4.10%) | 1.8 ± 0.5 | 1.8 ± 0.5 | 0.894 |
| Hb (g/L), Mean ± SD | 19 (4.10%) | 139.8 ± 17.9 | 139.7 ± 17.7 | 0.938 |
| Psoas CSA (cm²), Mean ± SD | 19 (4.10%) | 4.0 ± 1.2 | 4.0 ± 1.2 | 0.826 |
| Erector CSA (cm²), Mean ± SD | 19 (4.10%) | 7.8 ± 1.8 | 7.8 ± 1.8 | 0.854 |
| Multifidus CSA (cm²), Mean ± SD | 19 (4.10%) | 3.1 ± 0.7 | 3.1 ± 0.7 | 0.997 |
| Psoas fat (%), Mean ± SD | 19 (4.10%) | 13.3 ± 3.4 | 13.3 ± 3.4 | 0.914 |
| Erector fat (%), Mean ± SD | 19 (4.10%) | 19.5 ± 4.9 | 19.5 ± 4.8 | 0.985 |
| Multifidus fat (%), Mean ± SD | 19 (4.10%) | 24.8 ± 5.3 | 24.8 ± 5.3 | 0.948 |
| Length of hospital stay (days), Mean ± SD | 0 (0.00%) | 8.7 ± 1.1 | 8.7 ± 1.1 | 1 |
| Total CSA (cm²), Mean ± SD | 19 (4.10%) | 14.9 ± 3.5 | 14.8 ± 3.5 | 0.863 |
| Mean fat (%), Mean ± SD | 19 (4.10%) | 19.2 ± 4.0 | 19.2 ± 4.0 | 0.956 |
| Admission VAS, Mean ± SD | 17 (3.67%) | 5.8 ± 0.8 | 5.8 ± 0.8 | 0.902 |
| Discharge VAS, Mean ± SD | 18 (3.89%) | 3.4 ± 0.9 | 3.4 ± 0.9 | 0.947 |
| Admission JOA, Mean ± SD | 0 (0.00%) | 10.3 ± 1.2 | 10.3 ± 1.2 | 1 |
| Discharge JOA, Mean ± SD | 0 (0.00%) | 20.1 ± 0.8 | 20.1 ± 0.8 | 1 |
| 1-month JOA, Mean ± SD | 79 (17.06%) | 20.3 ± 1.9 | 20.3 ± 1.9 | 0.888 |
| 3-month JOA, Mean ± SD | 138 (29.81%) | 20.7 ± 2.2 | 20.5 ± 2.3 | 0.241 |
| Discharge Outcome, n (%) | 0 (0.00%) |  |  | 1 |
| Poor |  | 135 (29.2) | 135 (29.2) |  |
| Good |  | 328 (70.8) | 328 (70.8) |  |
| 1-month Outcome, n (%) | 79 (17.06%) |  |  | 0.917 |
| Poor |  | 138 (35.9) | 168 (36.3) |  |
| Good |  | 246 (64.1) | 295 (63.7) |  |
| 3-month Outcome, n (%) | 138 (29.81%) |  |  | 0.147 |
| Poor |  | 95 (29.2) | 158 (34.1) |  |
| Good |  | 230 (70.8) | 305 (65.9) |  |
| ASMI (kg/m2), Mean ± SD | 0 (0.00%) | 6.5 ± 1.4 | 6.5 ± 1.4 | 1 |
| Grip strength (kg), Mean ± SD | 30 (6.48%) | 27.5 ± 8.5 | 27.3 ± 8.4 | 0.733 |
| Gait speed (m/s), Mean ± SD | 0 (0.00%) | 1.1 ± 0.2 | 1.1 ± 0.2 | 1 |
| Main stenosis level, n (%) | 22 (4.75%) |  |  | 0.999 |
| L2/3 |  | 7 (1.6) | 7 (1.5) |  |
| L3/4 |  | 48 (10.9) | 51 (11.0) |  |
| L4/5 |  | 332 (75.3) | 349 (75.4) |  |
| L5/S1 |  | 54 (12.2) | 56 (12.1) |  |
| Schizas grade, n (%) | 22 (4.75%) |  |  | 0.996 |
| Grade B |  | 165 (37.4) | 172 (37.1) |  |
| Grade C |  | 194 (44.0) | 205 (44.3) |  |
| Grade D |  | 82 (18.6) | 86 (18.6) |  |
| Analgesic use, n (%) | 31 (6.70%) |  |  | 0.937 |
| No |  | 142 (32.9) | 150 (32.4) |  |
| Yes |  | 290 (67.1) | 313 (67.6) |  |
| Note: Normally distributed data are shown as mean ± SD; skewed CRP data are presented as median (Q1, Q3); categorical data are reported as count (%). P values test distribution differences between imputed and raw datasets.  Abbreviations and variable definitions are consistent with Table 1. | | | | |
|  |  |  |  |  |
