# Supplementary material for: Effect of sarcopenia and paraspinal muscle morphology on conservative treatment outcomes in lumbar spinal stenosis: a prospective cohort study
Source: Front Aging. 2026 Aug 26;7:1913710. doi: 10.3389/fragi.2026.1913710 (PMC13553900; doi:10.3389/fragi.2026.1913710)
Supplement: Supplementary file 4 [file Table2.docx]

| **Supplementary Table 2a. Comparison of descriptive statistics between unimputed and imputed data in the sarcopenia group (n=89)** | | | | |
| --- | --- | --- | --- | --- |
| Variables | Missing cases, n (%) | Before imputation | After imputation | P value |
| Sarcopenia, n (%) | 0 (0.00%) |  |  | 1.000 |
| No |  | 0 (0.0) | 0 (0.0) |  |
| Yes |  | 89 (100.0) | 89 (100.0) |  |
| Sex, n (%) | 0 (0.00%) |  |  | 1.000 |
| Male |  | 28 (31.5) | 28 (31.5) |  |
| Female |  | 61 (68.5) | 61 (68.5) |  |
| Age (y), Mean ± SD | 0 (0.00%) | 75.9 ± 6.8 | 75.9 ± 6.8 | 1.000 |
| Living status, n (%) | 9 (10.11%) |  |  | 1.000 |
| Living with others |  | 71 (88.8) | 79 (88.8) |  |
| Living alone |  | 9 (11.2) | 10 (11.2) |  |
| Hypertension, n (%) | 4 (4.49%) |  |  | 0.909 |
| No |  | 47 (55.3) | 51 (57.3) |  |
| Yes |  | 38 (44.7) | 38 (42.7) |  |
| DM, n (%) | 3 (3.37%) |  |  | 1.000 |
| No |  | 49 (57.0) | 50 (56.2) |  |
| Yes |  | 37 (43.0) | 39 (43.8) |  |
| Hyperlipidemia, n (%) | 9 (10.11%) |  |  | 0.890 |
| No |  | 53 (66.2) | 57 (64.0) |  |
| Yes |  | 27 (33.8) | 32 (36.0) |  |
| Smoking, n (%) | 2 (2.25%) |  |  | 1.000 |
| No |  | 56 (64.4) | 57 (64.0) |  |
| Yes |  | 31 (35.6) | 32 (36.0) |  |
| Alcohol, n (%) | 10 (11.24%) |  |  | 0.981 |
| No |  | 57 (72.2) | 63 (70.8) |  |
| Yes |  | 22 (27.8) | 26 (29.2) |  |
| BMI, Mean ± SD | 2 (2.25%) | 23.2 ± 3.4 | 23.2 ± 3.4 | 0.977 |
| TP (g/L), Mean ± SD | 3 (3.37%) | 66.7 ± 5.4 | 66.6 ± 5.4 | 0.908 |
| Alb (g/L), Mean ± SD | 3 (3.37%) | 38.3 ± 3.0 | 38.3 ± 3.0 | 0.892 |
| BUN (mmol/L), Mean ± SD | 0 (0.00%) | 6.2 ± 1.4 | 6.2 ± 1.4 | 1.000 |
| Cr (μmol/L), Mean ± SD | 0 (0.00%) | 69.2 ± 15.4 | 69.2 ± 15.4 | 1.000 |
| CRP (mg/L), Median(Q1, Q3) | 0 (0.00%) | 10.2 (5.5, 17.4) | 10.2 (5.5, 17.4) | 1.000 |
| WBC (10^9/L), Mean ± SD | 5 (5.62%) | 6.2 ± 1.4 | 6.2 ± 1.4 | 0.858 |
| NEUT (10^9/L), Mean ± SD | 5 (5.62%) | 3.5 ± 0.8 | 3.5 ± 0.8 | 0.900 |
| PLT (10^9/L), Mean ± SD | 5 (5.62%) | 206.1 ± 52.9 | 207.7 ± 52.3 | 0.840 |
| LYM (10^9/L), Mean ± SD | 5 (5.62%) | 1.8 ± 0.5 | 1.7 ± 0.5 | 0.681 |
| Hb (g/L), Mean ± SD | 5 (5.62%) | 138.8 ± 15.3 | 138.7 ± 15.1 | 0.964 |
| Psoas CSA (cm²), Mean ± SD | 4 (4.49%) | 2.9 ± 0.8 | 2.9 ± 0.8 | 0.945 |
| Erector CSA (cm²), Mean ± SD | 4 (4.49%) | 5.6 ± 1.1 | 5.6 ± 1.1 | 0.888 |
| Multifidus CSA (cm²), Mean ± SD | 4 (4.49%) | 2.3 ± 0.4 | 2.3 ± 0.4 | 0.952 |
| Psoas fat (%), Mean ± SD | 4 (4.49%) | 18.5 ± 3.2 | 18.5 ± 3.2 | 0.985 |
| Erector fat (%), Mean ± SD | 4 (4.49%) | 26.9 ± 4.2 | 26.8 ± 4.2 | 0.928 |
| Multifidus fat (%), Mean ± SD | 4 (4.49%) | 32.3 ± 3.6 | 32.4 ± 3.5 | 0.883 |
| Length of hospital stay (days), Mean ± SD | 0 (0.00%) | 9.5 ± 1.1 | 9.5 ± 1.1 | 1.000 |
| Total CSA (cm²), Mean ± SD | 4 (4.49%) | 10.8 ± 2.1 | 10.8 ± 2.1 | 0.912 |
| Mean fat (%), Mean ± SD | 4 (4.49%) | 25.9 ± 2.5 | 25.9 ± 2.5 | 0.979 |
| Admission VAS, Mean ± SD | 3 (3.37%) | 6.0 ± 0.8 | 5.9 ± 0.8 | 0.935 |
| Discharge VAS, Mean ± SD | 3 (3.37%) | 3.7 ± 0.9 | 3.7 ± 0.9 | 0.949 |
| Admission JOA, Mean ± SD | 0 (0.00%) | 9.8 ± 1.3 | 9.8 ± 1.3 | 1.000 |
| Discharge JOA, Mean ± SD | 0 (0.00%) | 19.6 ± 0.9 | 19.6 ± 0.9 | 1.000 |
| 1-month JOA, Mean ± SD | 12 (13.48%) | 17.9 ± 1.4 | 17.9 ± 1.3 | 0.990 |
| 3-month JOA, Mean ± SD | 33 (37.08%) | 18.0 ± 1.6 | 17.9 ± 1.6 | 0.821 |
| Discharge Outcome, n (%) | 0 (0.00%) |  |  | 1.000 |
| Poor |  | 57 (64.0) | 57 (64.0) |  |
| Good |  | 32 (36.0) | 32 (36.0) |  |
| 1-month Outcome, n (%) | 12 (13.48%) |  |  | 1.000 |
| Poor |  | 70 (90.9) | 81 (91.0) |  |
| Good |  | 7 (9.1) | 8 (9.0) |  |
| 3-month Outcome, n (%) | 33 (37.08%) |  |  | 0.916 |
| Poor |  | 46 (82.1) | 75 (84.3) |  |
| Good |  | 10 (17.9) | 14 (15.7) |  |
| ASMI (kg/m^2^), Mean ± SD | 0 (0.00%) | 5.4 ± 1.0 | 5.4 ± 1.0 | 1.000 |
| Grip strength (kg), Mean ± SD | 12 (13.48%) | 20.5 ± 5.9 | 20.6 ± 5.7 | 0.956 |
| Gait speed (m/s), Mean ± SD | 0 (0.00%) | 1.0 ± 0.1 | 1.0 ± 0.1 | 1.000 |
| Main stenosis level, n (%) | 9 (10.11%) |  |  | 1.000 |
| L2/3 |  | 1 (1.2) | 1 (1.1) |  |
| L3/4 |  | 7 (8.8) | 8 (9.0) |  |
| L4/5 |  | 63 (78.8) | 71 (79.8) |  |
| L5/S1 |  | 9 (11.2) | 9 (10.1) |  |
| Schizas grade, n (%) | 9 (10.11%) |  |  | 0.971 |
| Grade B |  | 24 (30.0) | 28 (31.5) |  |
| Grade C |  | 41 (51.2) | 44 (49.4) |  |
| Grade D |  | 15 (18.8) | 17 (19.1) |  |
| Analgesic use, n (%) | 11 (12.36%) |  |  | 1.000 |
| No |  | 20 (25.6) | 23 (25.8) |  |
| Yes |  | 58 (74.4) | 66 (74.2) |  |

| **Supplementary Table 2b.Comparison of descriptive statistics between unimputed and imputed data in the non-sarcopenia group (n=374)** | | | | |
| --- | --- | --- | --- | --- |
| Variables | Missing cases, n (%) | Before imputation | After imputation | P value |
| Sarcopenia, n (%) | 0 (0.00%) |  |  | 1.000 |
| No |  | 374 (100.0) | 374 (100.0) |  |
| Yes |  | 0 (0.0) | 0 (0.0) |  |
| Sex, n (%) | 0 (0.00%) |  |  | 1.000 |
| Male |  | 166 (44.4) | 166 (44.4) |  |
| Female |  | 208 (55.6) | 208 (55.6) |  |
| Age (y), Mean ± SD | 0 (0.00%) | 69.7 ± 6.2 | 69.7 ± 6.2 | 1.000 |
| Living status, n (%) | 36 (9.63%) |  |  | 1.000 |
| Living with others |  | 299 (88.5) | 330 (88.2) |  |
| Living alone |  | 39 (11.5) | 44 (11.8) |  |
| Hypertension, n (%) | 16 (4.28%) |  |  | 0.961 |
| No |  | 260 (72.6) | 270 (72.2) |  |
| Yes |  | 98 (27.4) | 104 (27.8) |  |
| DM, n (%) | 13 (3.48%) |  |  | 0.691 |
| No |  | 231 (64.0) | 233 (62.3) |  |
| Yes |  | 130 (36.0) | 141 (37.7) |  |
| Hyperlipidemia, n (%) | 19 (5.08%) |  |  | 0.913 |
| No |  | 233 (65.6) | 243 (65.0) |  |
| Yes |  | 122 (34.4) | 131 (35.0) |  |
| Smoking, n (%) | 13 (3.48%) |  |  | 0.990 |
| No |  | 217 (60.1) | 226 (60.4) |  |
| Yes |  | 144 (39.9) | 148 (39.6) |  |
| Alcohol, n (%) | 25 (6.68%) |  |  | 0.931 |
| No |  | 235 (67.3) | 254 (67.9) |  |
| Yes |  | 114 (32.7) | 120 (32.1) |  |
| BMI, Mean ± SD | 10 (2.67%) | 24.3 ± 3.1 | 24.2 ± 3.2 | 0.856 |
| TP (g/L), Mean ± SD | 26 (6.95%) | 68.4 ± 5.1 | 68.4 ± 5.1 | 0.926 |
| Alb (g/L), Mean ± SD | 26 (6.95%) | 40.4 ± 3.1 | 40.4 ± 3.2 | 0.991 |
| BUN (mmol/L), Mean ± SD | 14 (3.74%) | 6.3 ± 1.5 | 6.3 ± 1.5 | 0.857 |
| Cr (μmol/L), Mean ± SD | 14 (3.74%) | 71.0 ± 16.2 | 70.9 ± 16.0 | 0.954 |
| CRP (mg/L), Median(Q1, Q3) | 6 (1.60%) | 7.3 (5.0, 11.9) | 7.3 (5.0, 12.0) | 0.998 |
| WBC (10^9/L), Mean ± SD | 14 (3.74%) | 6.3 ± 1.6 | 6.4 ± 1.6 | 0.853 |
| NEUT (10^9/L), Mean ± SD | 14 (3.74%) | 3.7 ± 0.9 | 3.7 ± 0.9 | 0.917 |
| PLT (10^9/L), Mean ± SD | 14 (3.74%) | 200.4 ± 46.8 | 201.1 ± 46.4 | 0.831 |
| LYM (10^9/L), Mean ± SD | 14 (3.74%) | 1.7 ± 0.5 | 1.8 ± 0.5 | 0.748 |
| Hb (g/L), Mean ± SD | 14 (3.74%) | 140.1 ± 18.5 | 140.0 ± 18.3 | 0.950 |
| Psoas CSA (cm²), Mean ± SD | 15 (4.01%) | 4.2 ± 1.1 | 4.2 ± 1.1 | 0.826 |
| Erector CSA (cm²), Mean ± SD | 15 (4.01%) | 8.3 ± 1.6 | 8.3 ± 1.6 | 0.867 |
| Multifidus CSA (cm²), Mean ± SD | 15 (4.01%) | 3.3 ± 0.6 | 3.3 ± 0.6 | 0.960 |
| Psoas fat (%), Mean ± SD | 15 (4.01%) | 12.1 ± 2.1 | 12.1 ± 2.1 | 0.884 |
| Erector fat (%), Mean ± SD | 15 (4.01%) | 17.7 ± 3.0 | 17.7 ± 3.0 | 0.990 |
| Multifidus fat (%), Mean ± SD | 15 (4.01%) | 23.1 ± 3.9 | 23.1 ± 3.9 | 0.998 |
| Length of hospital stay (days), Mean ± SD | 0 (0.00%) | 8.5 ± 1.0 | 8.5 ± 1.0 | 1.000 |
| Total CSA (cm²), Mean ± SD | 15 (4.01%) | 15.8 ± 3.0 | 15.8 ± 3.0 | 0.871 |
| Mean fat (%), Mean ± SD | 15 (4.01%) | 17.6 ± 2.2 | 17.6 ± 2.2 | 0.962 |
| Admission VAS, Mean ± SD | 14 (3.74%) | 5.8 ± 0.8 | 5.8 ± 0.8 | 0.924 |
| Discharge VAS, Mean ± SD | 15 (4.01%) | 3.4 ± 0.9 | 3.4 ± 0.9 | 0.918 |
| Admission JOA, Mean ± SD | 0 (0.00%) | 10.4 ± 1.2 | 10.4 ± 1.2 | 1.000 |
| Discharge JOA, Mean ± SD | 0 (0.00%) | 20.2 ± 0.8 | 20.2 ± 0.8 | 1.000 |
| 1-month JOA, Mean ± SD | 67 (17.91%) | 20.9 ± 1.5 | 20.8 ± 1.6 | 0.947 |
| 3-month JOA, Mean ± SD | 105 (28.07%) | 21.3 ± 1.9 | 21.1 ± 2.0 | 0.360 |
| Discharge Outcome, n (%) | 0 (0.00%) |  |  | 1.000 |
| Poor |  | 78 (20.9) | 78 (20.9) |  |
| Good |  | 296 (79.1) | 296 (79.1) |  |
| 1-month Outcome, n (%) | 67 (17.91%) |  |  | 0.801 |
| Poor |  | 68 (22.1) | 87 (23.3) |  |
| Good |  | 239 (77.9) | 287 (76.7) |  |
| 3-month Outcome, n (%) | 105 (28.07%) |  |  | 0.257 |
| Poor |  | 49 (18.2) | 83 (22.2) |  |
| Good |  | 220 (81.8) | 291 (77.8) |  |
| ASMI (kg/m^2^), Mean ± SD | 0 (0.00%) | 6.8 ± 1.3 | 6.8 ± 1.3 | 1.000 |
| Grip strength (kg), Mean ± SD | 18 (4.81%) | 29.1 ± 8.3 | 29.0 ± 8.2 | 0.871 |
| Gait speed (m/s), Mean ± SD | 0 (0.00%) | 1.1 ± 0.1 | 1.1 ± 0.1 | 1.000 |
| Main stenosis level, n (%) | 13 (3.48%) |  |  | 1.000 |
| L2/3 |  | 6 (1.7) | 6 (1.6) |  |
| L3/4 |  | 41 (11.4) | 43 (11.5) |  |
| L4/5 |  | 269 (74.5) | 278 (74.3) |  |
| L5/S1 |  | 45 (12.5) | 47 (12.6) |  |
| Schizas grade, n (%) | 13 (3.48%) |  |  | 0.983 |
| Grade B |  | 141 (39.1) | 144 (38.5) |  |
| Grade C |  | 153 (42.4) | 161 (43.0) |  |
| Grade D |  | 67 (18.6) | 69 (18.4) |  |
| Analgesic use, n (%) | 20 (5.35%) |  |  | 0.948 |
| No |  | 122 (34.5) | 127 (34.0) |  |
| Yes |  | 232 (65.5) | 247 (66.0) |  |
