# Supplementary material for: Effect of sarcopenia and paraspinal muscle morphology on conservative treatment outcomes in lumbar spinal stenosis: a prospective cohort study
Source: Front Aging. 2026 Aug 26;7:1913710. doi: 10.3389/fragi.2026.1913710 (PMC13553900; doi:10.3389/fragi.2026.1913710)
Supplement: Supplementary file 5 [file Table3.docx]

| **Supplementary Table 3. Skewness and kurtosis of continuous and ordinal variables for normality assessment** | | | |
| --- | --- | --- | --- |
| Variable | \|Skewness\| | \|Kurtosis\| | Normality |
| Age (y) | 0.318 | 0.595 | Approximately normal |
| BMI | 0.138 | 0.429 | Approximately normal |
| TP (g/L) | 0.141 | 0.073 | Approximately normal |
| Alb (g/L) | 0.114 | 0.186 | Approximately normal |
| BUN (mmol/L) | 0.208 | 0.081 | Approximately normal |
| Cr (μmol/L) | 0.571 | 0.118 | Approximately normal |
| CRP (mg/L) | 1.042 | 0.7 | Skewed distribution |
| WBC (10^9/L) | 0.382 | 0.108 | Approximately normal |
| NEUT (10^9/L) | 0.351 | 0.189 | Approximately normal |
| PLT (10^9/L) | 0.09 | 0.576 | Approximately normal |
| LYM (10^9/L) | 0.729 | 0.577 | Approximately normal |
| Hb (g/L) | 0.056 | 0.233 | Approximately normal |
| Psoas CSA (cm²) | 0.436 | 0.772 | Approximately normal |
| Erector CSA (cm²) | 0.226 | 0.546 | Approximately normal |
| Multifidus CSA (cm²) | 0.129 | 0.263 | Approximately normal |
| Psoas fat (%) | 0.637 | 0.926 | Approximately normal |
| Erector fat (%) | 0.999 | 0.853 | Approximately normal |
| Multifidus fat (%) | 0.432 | 0.594 | Approximately normal |
| Length of hospital stay (days) | 0.398 | 0.259 | Approximately normal |
| Total CSA (cm²) | 0.164 | 0.822 | Approximately normal |
| Mean fat (%) | 0.993 | 0.231 | Approximately normal |
| Admission VAS | 0.114 | 0.214 | Approximately normal |
| Discharge VAS | 0.129 | 0.002 | Approximately normal |
| Admission JOA | 0.149 | 0.21 | Approximately normal |
| Discharge JOA | 0.317 | 0.128 | Approximately normal |
| 1-month JOA | 0.155 | 0.451 | Approximately normal |
| 3-month JOA | 0.145 | 0.539 | Approximately normal |
| ASMI (kg/m2) | 0.426 | 1 | Approximately normal |
| Grip strength (kg) | 0.312 | 1.062 | Approximately normal |
| Gait speed (m/s) | 0.041 | 0.119 | Approximately normal |
| Criterion: \|Skewness\| < 1 and \|excess Kurtosis\| < 2, approximately normal; otherwise skewed distribution. | | | |
