# Supplementary material for: Effect of sarcopenia and paraspinal muscle morphology on conservative treatment outcomes in lumbar spinal stenosis: a prospective cohort study
Source: Front Aging. 2026 Aug 26;7:1913710. doi: 10.3389/fragi.2026.1913710 (PMC13553900; doi:10.3389/fragi.2026.1913710)
Supplement: Supplementary file 6 [file Table6.docx]

| **Supplementary Table 6. Multicollinearity assessment via variance inflation factor (VIF)** | | |
| --- | --- | --- |
| Variable | VIF | Collinearity |
| Sarcopenia | 1.209 | None |
| Sex | 1.013 | None |
| Age | 1.172 | None |
| Albumin | 1.09 | None |
| Notes: Collinearity was defined as VIF ≥ 5; VIF < 5 indicates no significant multicollinearity between variables. | | |
