# Supplementary material for: Effect of sarcopenia and paraspinal muscle morphology on conservative treatment outcomes in lumbar spinal stenosis: a prospective cohort study
Source: Front Aging. 2026 Aug 26;7:1913710. doi: 10.3389/fragi.2026.1913710 (PMC13553900; doi:10.3389/fragi.2026.1913710)
Supplement: Supplementary file 7 [file Table7.docx]

| **Supplementary Table 7. Sensitivity analysis for the association between sarcopenia and treatment outcome** | | |
| --- | --- | --- |
| Model | Effect estimate (95% CI) | P value |
| Complete-case analysis (discharge outcome) | OR=0.21 (0.12 to 0.37) | <0.001 |
| Analysis using length of hospital stay as outcome | β=1.01 (0.76 to 1.26) | <0.001 |
| Notes: All models were adjusted for sex, age, and albumin. Complete-case analysis included only patients with complete data for all covariates (n=384). For length of hospital stay, linear regression was used, and the effect estimate is the unstandardized coefficient β. | | |
|  |  |  |
|  |  |  |
