# Supplementary material for: Effect of sarcopenia and paraspinal muscle morphology on conservative treatment outcomes in lumbar spinal stenosis: a prospective cohort study
Source: Front Aging. 2026 Aug 26;7:1913710. doi: 10.3389/fragi.2026.1913710 (PMC13553900; doi:10.3389/fragi.2026.1913710)
Supplement: Supplementary file 8 [file Table8.docx]

| **Supplementary Table 8. Firth-corrected logistic regression models for sarcopenia predicting short-term adverse outcomes** | | | |
| --- | --- | --- | --- |
| Model | Adjusted covariates | OR (95% CI) for1-month outcome | OR (95% CI) for 3-month outcome |
| Multivariable model | sex + age + albumin | 0.022 (0.008–0.054) | 0.058 (0.027–0.117) |
| Simplified model 1 | age | 0.045 (0.020–0.092) | 0.079 (0.041–0.146) |
| Simplified model 2 | sex | 0.015 (0.005–0.036) | 0.040 (0.019–0.079) |
| Simplified model 3 | albumin | 0.033 (0.014–0.066) | 0.054 (0.028–0.098) |
