# Supplementary material for: Effect of sarcopenia and paraspinal muscle morphology on conservative treatment outcomes in lumbar spinal stenosis: a prospective cohort study
Source: Front Aging. 2026 Aug 26;7:1913710. doi: 10.3389/fragi.2026.1913710 (PMC13553900; doi:10.3389/fragi.2026.1913710)
Supplement: Supplementary file 9 [file Table9.docx]

| **Supplementary Table 9. Associations between sarcopenia-related indicators and discharge outcome in univariate and fully adjusted logistic regression models** | | | | |
| --- | --- | --- | --- | --- |
|  | **Unadjusted** | | **Fully adjusted** | |
| **Continuous variables** | OR (95%CI) | P value | OR (95%CI) | P value |
| Gait speed (m/s) | 17.58 (4.33~71.36) | <0.001 | 1.45 (0.25~8.20) | 0.677 |
| Grip strength (kg) | 1.12 (1.09~1.16) | <0.001 | 1.13 (1.06~1.20) | <0.001 |
| ASMI (kg/m2) | 1.99 (1.63~2.42) | <0.001 | 2.01 (1.36~2.97) | <0.001 |
| **Categorical variables** |  |  |  |  |
| Gait speed (Normal vs. Slow) | 2.15 (1.38~3.35) | <0.001 | 1.28 (0.78~2.11) | 0.321 |
| Grip strength (Normal vs. Low) | 5.62 (3.39~9.30) | <0.001 | 3.44 (1.98~5.99) | <0.001 |
| ASMI (Normal vs. Low) | 2.70 (1.77~4.10) | <0.001 | 1.98 (1.25~3.14) | 0.004 |
| Note: Unadjusted = univariate logistic regression; fully adjusted models controlled for age, sex and albumin. Categorical cutoffs for sarcopenia indicators followed 2019 AWGS criteria. | | | | |
